# Generative deep learning for foundational video translation in ultrasound

**DOI:** 10.1038/s41598-026-47777-z

**Published:** 2026-04-16

**Authors:** Nikolina Tomic, Roshni Bhatnagar, Sarthak Jain, Connor Lau, Tien-Yu Liu, Laura Gambini, Rima Arnaout

**Affiliations:** 1 Department of Medicine, Division of Cardiology, Bakar Computational Health Sciences Institute, University of California, San Francisco, 521 Parnassus Avenue, San Francisco, CA 94143 USA; 2 UCSF-UC Berkeley Joint Program in Computational Precision Health, Department of Radiology and Pediatrics Center for Intelligent Imaging, University of California, San Francisco, San Francisco CA, 94143 USA

**Keywords:** Generative adversarial networks, Image synthesis, Medical imaging, Ultrasound, Video translation, Computational biology and bioinformatics, Health care, Mathematics and computing, Medical research

## Abstract

For deep learning (DL) to realize its potential for medical image interpretation, attention to dataset content is critical. Ultrasound presents a particular challenge because, in addition to many views and structures, it includes several sub-modalities–such as grayscale and color flow doppler (CFD)–that are often imbalanced or confounding in clinical datasets. Image translation could help, but it has not yet succeeded in noisy ultrasound. Here, we develop generative video translation for CFD-to-grayscale ultrasound. We leveraged pixel-wise, adversarial, and perceptual losses to synthesize anatomically faithful, realistic-looking ultrasound. Average SSIM between synthetic and ground-truth videos was 0.91 ± 0.04. Synthetic videos performed indistinguishably in DL classification (F1-score between real and synthetic, 0.93–0.95) and segmentation tasks (average Dice between real and synthetic segmentations, 0.97 ± 0.03). Blinded clinician accuracy in distinguishing real vs. synthetic videos was 54 ± 6%, indicating realism. Although trained only on heart videos, the model worked on ultrasound spanning clinical domains (average SSIM 0.91 ± 0.05), demonstrating foundational abilities. Applying generative translation to real-world CFD imaging recovered over seven percent more data for a clinical DL task. Together, these data expand the utility of retrospective imaging, advance rigor in medical synthetic data evaluation, and augment the dataset design toolbox for medical imaging.

## Introduction

Ultrasound is one of the highest-volume medical imaging modalities worldwide and is critical to diagnosis and management across organ systems. Deep learning (DL) has numerous use cases in ultrasound, from cardiac imaging^1–5^ to fetal screening^6,7^ to abdominal evaluation^8^. DL can expand access to expert image interpretation^6,7^ extract additional information from existing images^3,9,10^ and improve efficiency^11,12^ of interpretation, segmentation, and other tasks^1,13,14^.

Growing experience has shown the importance of curating training and test datasets^15–17^ for DL models. For example, it is increasingly clear that proper dataset design must consider overall dataset diversity, including both inter- and intra-class variation, above and beyond the simple metrics of size and class balance^18–20^. Without dataset design and augmentation strategies that recognize and mitigate potential inter- and intra-class confounders, DL models may fail to learn clinically relevant features for the task at hand, potentially overfitting on artifacts or confounding features that happen to differ among classes.

**Fig. 1:**
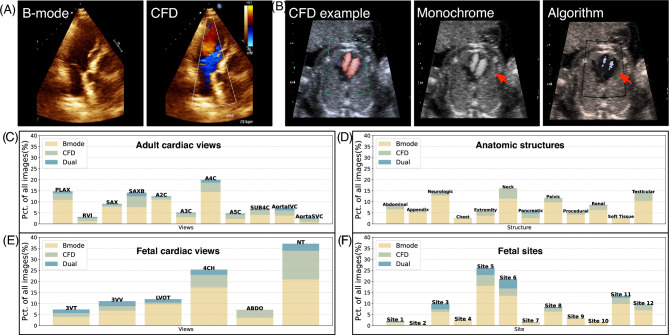
B-mode and color flow doppler (CFD) offer different information, belie simple translation methods, and are unbalanced in datasets. (**A**) Example B-mode and corresponding CFD ultrasound. (**B**) Monochrome rendering makes the CFD signal grey (red arrow) instead of matching the blood pool. Algorithmically detecting colored pixels and replacing them with grayscale values has several problems, including retaining the CFD region of interest box, imperfect thresholds for colored pixels, and replacement greyscale values that do not match the color or noise profile of the ultrasound image at hand (red arrow). Furthermore, anatomic structures underneath the original color signal are not reconstructed. The prevalence of B-mode, CFD, and dual acquisitions is imbalanced and varies across typical clinical views (**C**, **E**); different anatomic structures (**D**); and even across different clinical sites (**F**). *PLAX* parasternal long axis, *RVI* right ventricular inflow, *SAX* short axis mid or mitral, *SAXB* basal short axis, *A2C* apical two chamber, *A3C* apical three chamber/long axis, *A4C/4CH* four chamber, *A5C* apical five chamber, *SUB4C* subcostal four-chamber, *AortaIVC* abdominal aorta or subcostal inferior vena cava, *AortaSVC* aortic arch or superior vena cava, *3VT* three-vessel trachea, *3VV* three-vessel view, *LVOT* left-ventricular outflow tract, *ABDO* abdomen, *NT* non-target.

Ultrasound exemplifies these challenges, in large part because it comprises several sub-modalities including B-mode, color flow Doppler (CFD) (Fig. 1A), m-mode, spectral Doppler, and more^21^. In particular, B-mode and CFD both display two-dimensional images/videos of anatomic structures and are therefore likely to be considered as a single modality. However, the use of CFD varies greatly by sonographer, protocol, and views and anatomic structures being imaged, leading to an imbalanced presence of CFD within classes of interest for many important clinical tasks^1,6^(Fig. 1C-F). Thus, the presence of CFD signals in an image can easily be misconstrued as a feature of interest in classification, segmentation, or other tasks. At the same time, avoiding this potentially confounding variable by removing all CFD images from the dataset discards valuable data and can worsen class imbalance.

Several approaches have been explored to address such inter-domain variability. Domain adaptation methods^22^ aim to make models trained on one domain generalize to another domain by aligning their feature distributions. However, these approaches can be limited when structural or textural differences between domains are substantial–as in ultrasound, where color flow overlays both content and texture. Instead, translating between CFD and B-mode would be a useful way to balance presence of CFD among classes without discarding data. Removing the CFD signal from an image would seem to be a simple task, but in reality belies simple algorithmic methods due to variable CFD color palettes, variable B-mode color maps (chromamaps), color mixing, variable gain as well as noise profiles in the underlying ultrasound image, and obfuscation of underlying anatomic structures (Fig. 1B).

DL-based image (or video) translation can potentially overcome these challenges. Image translation has been applied to medical imaging in various cases, such as between MRI sub-modalities^23,24^, MRI motion correction, PET denoising, and PET-CT translation^25^. However, translation remains a challenge in ultrasound due to its high spatial noise. Furthermore, translating CFD to B-mode also requires reconstruction of anatomic structures covered by the CFD signal. Inpainting missing or degraded image regions has been widely studied for natural images^26–29^, but in the medical domain, has largely remained limited to inpainting uniformly-shaped, artificially generated patches on individual frames^30,31^. This diverges from real-world scenarios, where missing or occluded regions are irregular in shape and size and correspond to complex anatomic structures rather than simple artificial masks. Importantly, real-world ultrasound is in video format, where inpainting not only requires generating plausible content within each frame but also maintaining temporal consistency across consecutive frames, making the task substantially more challenging than image inpainting^32^.

CFD to B-mode translation must therefore balance style transfer, content preservation, realism, and reconstruction of free-form regions with anatomic structures that are not seen in the CFD image/video. Synthetic images/videos must be robust to rigorous computational and clinical evaluation^33^. Ideally, the approach should work effectively on ultrasounds from all organ systems, even if not explicitly trained on them.

Previous methods have addressed some of the above needs, but primarily for photographic or natural imaging and often in a piecewise fashion. For example, generative adversarial networks (GANs) have been used for image and video translation^34^. Diffusion-based models may offer improved synthesis quality, although often at the expense of substantially greater training data requirements that are typically unavailable for ultrasound imaging^35,36^. Standard GAN frameworks consist of a generator and a discriminator trained using pixel-wise and adversarial losses; however, when translation also requires inpainting, these objectives alone are insufficient to accurately reconstruct missing regions while preserving visual, semantic, and anatomical consistency with the surrounding image^26–28,30,31^.

To attempt to address these limitations, prior work introduced non-adversarial objectives–such as perceptual, style, and feature-matching losses–to guide networks toward realistic and contextually consistent reconstructions^37^. Gated convolutions have been proposed^25^, which dynamically learn spatial masks to emphasize valid contextual regions during reconstruction. While effective at enforcing structural coherence, gated convolution–based networks often underperform in recovering fine-grained texture, motivating many state-of-the-art approaches to adopt two-stage pipelines^32^ in which a secondary network is used to refine high-frequency details^28,31^.

Despite these advances, no existing method sufficiently addresses the combined challenges of ultrasound video translation, including limited training data, speckle-dominated texture, strict anatomical constraints, and the need for clinically meaningful outputs. To address these limitations and provide a practical tool for CFD-to-B-mode ultrasound translation, we develop a generative deep learning framework for video-to-video translation that explicitly aligns architectural and training strategies with the characteristics of ultrasound imaging.

Here, we develop a two-stage generative framework that directly addresses the tradeoff between anatomical fidelity and texture realism by leveraging pixel-wise, adversarial, and perceptual losses to reconstruct anatomic structures while synthesizing realistic ultrasound textures. We test this approach across multiple anatomic structures, including those not seen during training. We extensively evaluate pipeline results, combining quantitative image metrics, functional deep learning tasks, and clinician-based perceptual assessment. Finally, we show real-world applicability of the approach by improving the diversity and balance of training data for congenital heart disease detection.

## Results

**Fig. 2:**
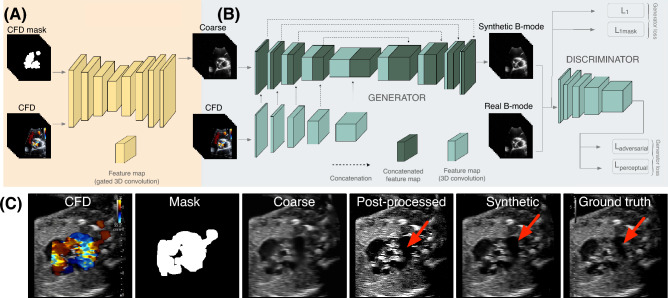
Pipeline. (**A**) The coarse network inputs the CFD video and a mask of the CFD region and uses gated convolutions to adaptively leverage spatially relevant features. (**B**) The refinement GAN combines L1 loss with adversarial and perceptual losses derived from the discriminator. (**C**) Example outputs from each step in the pipeline. Red arrows highlight how post-processing of the coarse output helps emphasize subtle anatomic features. Ground-truth shown for comparison.

We developed a generative deep learning pipeline to translate CFD ultrasound video to B-mode (Fig. 2, Methods). We trained and validated on 54,975 videos across fetal and adult ultrasounds with real-world clinical heterogeneity. We tested on 8370 videos (2866 adult and 5504 fetal, Table S1) as well as ultrasound from several other clinical domains.

**Fig. 3:**
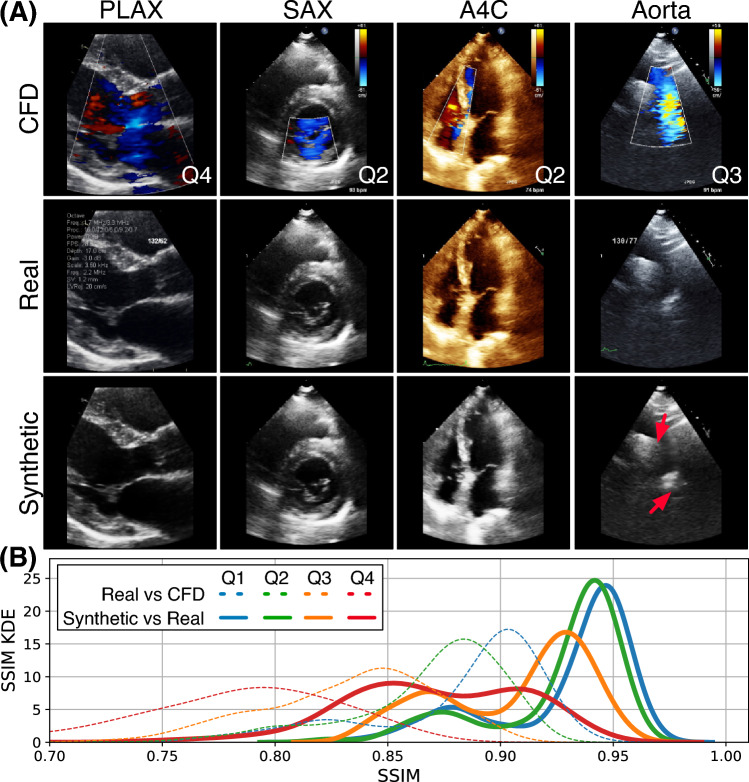
GAN pipeline generates realistic synthetic B-mode videos with high structural similarity for adult cardiac ultrasound. (**A**) Examples showing CFD, corresponding ground-truth B-mode, and synthetic B-mode. Anatomic structures underneath the color signal are reconstructed (red arrows) across quartiles of CFD coverage (indicated in lower right-hand corner of each CFD example). (**B**) SSIM kernel density estimates (KDE) comparing ground-truth B-mode to CFD (dashed line) and to synthetic B-mode (solid line). Q1 represents videos with the lowest CFD coverage (see Methods), and Q4 the highest. *PLAX* parasternal long axis, *SAX* short axis, *A4C* apical 4-chamber, *Aorta* aortic arch view.

**Fig. 4:**
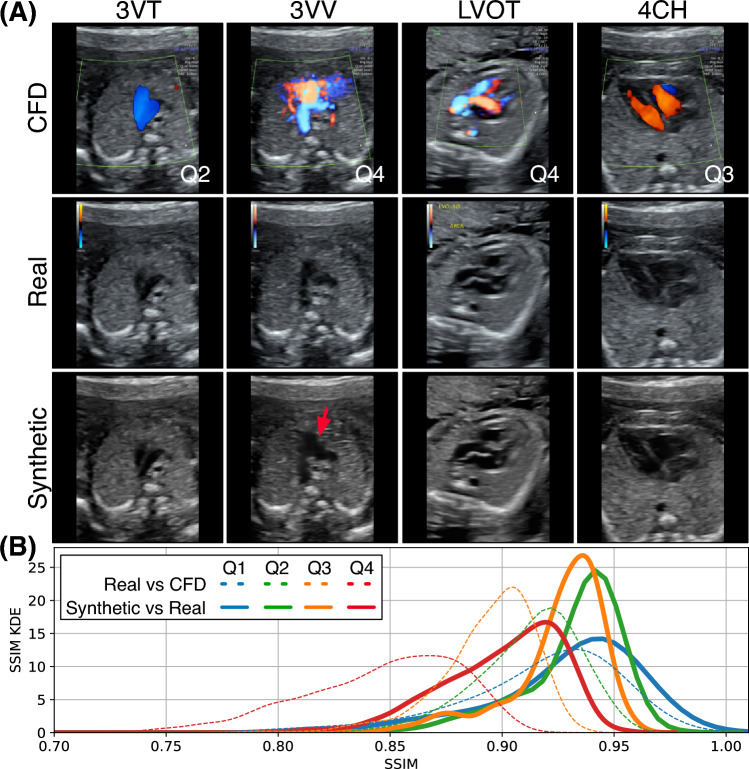
GAN pipeline also generates realistic B-mode videos for fetal ultrasound. (**A**) Examples, with quartiles of CFD coverage noted. In some examples with high CFD coverage, some fine structures, such as the septation between the pulmonary artery and the aorta in the 3VV view (red arrow), are less clear in the synthetic B-mode compared to ground truth. (**B**) SSIM kernel density estimates (KDE) comparing ground-truth B-mode to CFD (dashed line) and to synthetic B-mode (solid line). *3VT* 3-vessel trachea, *3VV* 3-vessel view, *LVOT* left-ventricular outflow tract, 4CH axial 4-chamber.

Overall, the structural similarity index measure (SSIM) between ground-truth B-mode frames and their synthetic counterparts was 0.91 ± 0.04 (0.1 - 0.98, n = 8370, Figs.3 and 4). The Fréchet inception distance (FID) was 36.

We evaluated synthetic videos for temporal consistency by calculating the mean absolute error (MAE) between consecutive frames warped via optical flow^38^. The mean temporal warping error for synthetic videos was $4.84 \pm 2.63$, which was statistically indistinguishable from that of real videos $4.89 \pm 2.43$ ($p = 0.16$, Welch’s t-test, $n = 8630$, Fig. S1). This confirms that the model replicates natural temporal dynamics and achieves high-quality motion synthesis without introducing artificial flickering. We provided more details in Supplementary Material.

**Fig. 5:**
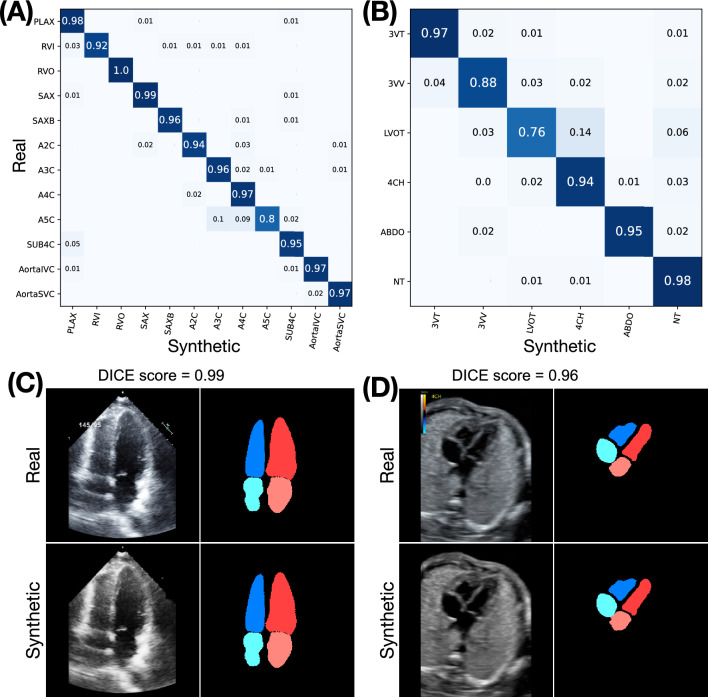
Zero-shot performance on several anatomic structures entirely held out during model training produces similarly realistic synthetic videos, suggesting foundational capabilities for video translation. (**A**) Examples of CFD frames, corresponding real (ground-truth) B-mode frames, and synthetic B-mode frames across various anatomic structures not represented in model training. The underlying anatomy is mostly reconstructed, but some artifacts are observed (red arrow). (**B**) SSIM kernel density estimates (KDE) comparing B-mode (ground truth) to CFD input data (dashed line) and B-mode to synthetic data (solid line). CFD videos are divided into quartiles (Q) of CFD coverage.

Synthetic videos were then evaluated using several functional tests. The performance of synthetic videos in benchmark view classification^4,6^ and segmentation tasks^1^ was statistically similar to that of real ones (Fig. 5). Clinical experts asked to distinguish real videos from synthetic ones achieved only 54 ± 6% accuracy overall (range, 42–61%)–no better than random chance.

**Fig. 6:**
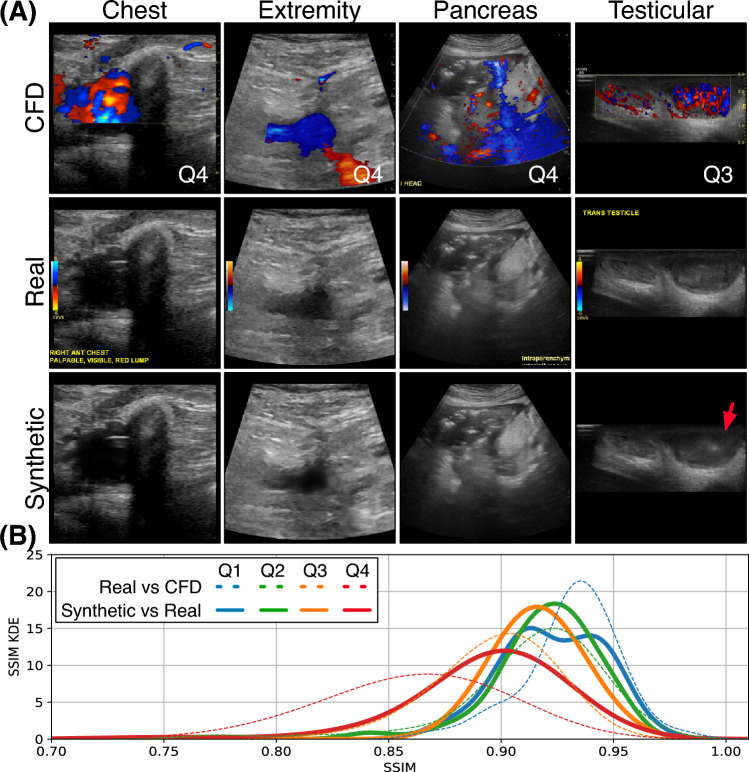
Functional testing of synthetic videos in deep learning benchmarks. Confusion matrices comparing adult (**A**) and fetal (**B**) view classification on real vs. synthetic B-mode. Examples of semantic segmentation on real vs. synthetic B-mode from adult (**C**) and fetal (**D**) datasets. *PLAX* parasternal long axis, *RVI* right ventricular inflow, *RVO* RV outflow, *SAX* short axis, *SAXB* basal short axis, *A2C* apical two chamber, *A3C* apical three chamber/long axis, *A4C/4CH* four chamber, *A5C* apical five chamber, *SUB4C* subcostal four-chamber, *AortaIVC* abdominal aorta/inferior vena cava, *AortaSVC* aortic arch/superior vena cava, *3VT* three-vessel trachea, *3VV* three-vessel view, *LVOT* left-ventricular outflow tract, *ABDO* abdomen, *NT* non-target.

Finally, to assess the foundational ability of our approach, we performed zero-shot evaluation on ultrasound exam types the model was not trained on and achieved an average SSIM of 0.91 ± 0.05 (0.43–0.97, n = 260, Fig. 6, Table S2). Taken together, these results indicate realistic, clinically relevant video-to-video translation among ultrasound sub-modalities.

### Structural similarity of synthetic video to ground truth is high

A minority of ultrasound acquisitions have simultaneously acquired B-mode and CFD videos. We leveraged these dual ultrasound acquisitions to train the GAN pipeline in a supervised manner, where the B-mode panel served as the ground truth. To ensure balanced learning across varying levels of CFD coverage, we quantified the proportion of CFD coverage within each video and divided the data into color quartiles (Q1–Q4). The model was guided to attend to CFD regions and trained in a two-step pipeline: a coarse network performing anatomic reconstruction, followed by a refinement network guiding style and texture.

We compared SSIM values between real B-mode and CFD image pairs, and between real B-modes and their synthetic B-mode counterparts. The resulting Wasserstein metrics showed notable SSIM shifts across adult, fetal, and other anatomic datasets (0.07, 0.03, and 0.02; Table S3), indicating that the synthetic B-mode outputs differ from the CFD inputs and more closely resemble the B-mode ground truth.

Since greater CFD coverage corresponds to a larger portion of the video frames requiring reconstruction, we analyzed performance across the previously defined quartiles (examples in Fig. 3, 4 and5; see also Methods, Estimating CFD Masks). As expected, higher CFD coverage was associated with slightly lower average SSIM yet higher Wasserstein distance. This was most prominently observed in Q4, which exhibited the largest deviation from input CFD videos.

### Synthetic videos performed indistinguishably in benchmark DL tasks

We also evaluated the performance of synthetic B-mode vs. real B-mode videos in two benchmark DL tasks–view classification and semantic segmentation–on two distinct datasets: adult and fetal ultrasound. We leveraged data encompassing 12 adult cardiac views and 6 fetal views, including screening ultrasound examinations as well as fetal cardiac ultrasound (see Fig.6 and Methods). We adopted previously developed deep learning models trained exclusively on real data^1,4,6,7^.

The F1 score on real B-mode videos (n = 2239) in an adult view classifier^4^ was 0.9, while the F1 score using synthetic B-mode videos was 0.89. There was no statistical difference among per-class F1 scores between real and synthetic videos (p = 0.68). The overall F1 score on real B-mode videos (n = 2203) in a fetal view classifier^6^ was 0.8, while the F1 score using synthetic B-mode videos was 0.79. As with the adult view classification task, there was no statistical difference among per-class F1-scores between real and synthetic videos (p = 0.81). Confusion matrices comparing inference of real vs synthetic videos for each task (Fig. 6A,B) had F1 scores of 0.95 and 0.93 for adult and fetal comparisons, respectively.

We then leveraged benchmark DL models for semantic segmentation for adult and fetal ultrasound^1,6^ (Fig. 5C,D). We compared the segmentation from real frames with segmentations from their synthetic B-mode counterparts. The average Dice score per class (excluding background) was 0.97 ± 0.03, for adult frames (range 0.87–0.99, n = 99) and 0.9 ± 0.16 for fetal frames (range 0.1–0.99, n = 171).

Taken together, these data show that real and synthetic videos are indistinguishable to benchmark DL models.

### Clinicians found real and synthetic videos indistinguishable

Beyond SSIM and functional testing in DL models, we evaluated human perception of synthetic videos using clinical experts.

*Human perception on clinical view classification* Clinical experts in adult (n = 6) and fetal (n = 8) ultrasound were asked to perform view classification–a common task in clinical practice– while blinded to the fact that some videos were synthetic. A post-hoc analysis of human view classification showed indistinguishable performance on real vs synthetic videos (p = 0.26 and p = 0.27 for adult and fetal, respectively). Table 1 details this performance.

**Table 1:** Performance of clinical experts on three tasks and three datasets.

Task	Dataset	Data origin	F1, mean ± std (range)	*P* value
Single video:	Adult	Real*	0.73 ± 0.10 (0.60–0.88)	0.26
		Synthetic*	0.81 ± 0.11 (0.66–0.91)	
“Classify by view"	Fetal	Real*	0.54 ± 0.12 (0.41–0.74)	0.27
		Synthetic*	0.46 ± 0.07 (0.35–0.54)	
	Other	Real*	0.71 ± 0.10 (0.51–0.82)	0.06
		Synthetic*	0.82 ± 0.05 (0.74–0.87)	

Task	Dataset	Data origin	Accuracy, mean ± std (range)	*P* value
Single	Adult	Real or synthetic	0.54 ± 0.06 (0.46–0.61)	-
video:	Fetal	Real or synthetic	0.57 ± 0.03 (0.53–0.6)	-
“Real or synthetic?”	Other	Real or synthetic	0.52 ± 0.1 (0.41–0.65)	-
Pair of	Adult	Real and synthetic	0.47 ± 0.05 (0.42–0.53)	-
videos:	Fetal	Real and synthetic	0.55 ± 0.05 (0.49–0.61)	-
“choose real”	Other	Real and synthetic	0.79 ± 0.05 (0.72–0.84)	-

*Clinicians were shown a mixture of real and synthetic images in a blinded fashion; analysis was performed post-hoc

*Human perception directly regarding video realism* Separately, clinicians (n = 3 for each adult task, n = 4 for each fetal task) were asked to identify if a given video was real or synthetic (i) when given one video at a time, and (ii) when given a pair of videos in which they are told one is real, and one is synthetic.

On the first task, accuracy was 54% for adult and 57% for fetal videos (where 50% accuracy represents random chance). On the second task, in which paired examples of real and fake videos were provided, accuracy was 47% for adult, and 55% for fetal videos. Overall, accuracy was 54 ± 6% (range, 42–61%). Table 1 details this performance by task and dataset.

*Correlation between human perception and quantitative similarity metric* Independently, three participants were asked to identify whether each synthetic video (n = 100) was real or synthetic, and for each sample, a fool rate coefficient was computed representing the proportion of subjects who perceived it as real. We analyzed the correlation between fool rate and SSIM between real B-mode and synthetic video. R² was 0.013 for adult and 0.168 for fetal data within the observed SSIM range (0.92 ± 0.03 range 0.83–0.96 for adult; 0.93 ± 0.03, range 0.84–0.98 for fetal).

### Balancing fetal ultrasound training datasets with synthetic data

Frames extracted from synthetic videos were also used to improve yield, class balance, and diversity^11^ of fetal imaging data used for clinical congenital heart disease (CHD) detection from B-mode imaging^6^. Specifically, 2,739,914 frames from 8,399 ultrasound exams (73,739 video clips in total) were recovered through our generative translation approach.

Previously, we had found that most diversity in fetal imaging data resides at the video clip level^11^; generative translation recovered 5.7% more video clips for clinical model training. These data included imaging from eight geographical regions (Fig. 1F). By recovering more B-mode imaging through our generative translation pipeline, the proportion of available data from the most under-represented state increased by 49% (Fig. S2). Finally, regarding disease distribution (normal vs. congenital heart disease), generative translation recruited significantly 20% more video clips from the most underrepresented group of CHD lesions (Fig. S2). (Of note, the above impacts of generative translation in improving clinical imaging dataset quality and content^39^ are significant underestimates, because without a powerful image translation pipeline previously, we have systematically avoided collecting CFD imaging to date.)

### Zero-shot performance on ultrasound covering other anatomies

To access the foundational ability of our approach, we performed zero-shot evaluation on 260 additional ultrasound exams (Fig. 1D, Table S2) spanning 11 different anatomies. These ultrasound exams correspond to anatomic structures that were entirely unseen by the model during training. Across this diverse dataset, average SSIM was 0.91 ± 0.05 (0.43–0.97, n = 260) and the FID was 78. After visual assessment, we found that the synthetic videos generally align well with real B-mode videos (Fig. 5).

Radiologists specializing in ultrasound were asked to classify anatomy (n = 6) and complete two tasks (n = 3 for each task) to determine whether each video (n = 100 for each task) was real or synthetic, as above. A post-hoc analysis of anatomy classification showed indistinguishable performance on real vs synthetic videos (with a trend toward significance, p = 0.06). On the tasks to identify if a given video was real or synthetic, accuracy was 52% when presented single video, and 79% when presented a pair of videos (Table 1).

### A/B testing demonstrates pipeline’s value over alternative methods

We evaluated the impact on synthetic video quality of (i) the two-stage, coarse-to-fine pipeline compared to standard one-step GANs and (ii) the use of post-processing between the coarse and the refine steps of the pipeline. For each head-to-head comparison, blinded participants ($n=3$ per dataset) assessed 200 randomly chosen videos (100 adult, 100 fetal) for perceptual quality.

*Comparison with baseline GAN* We trained a standard one-step GAN (inspired by^34^, more details available in Methods, A/B testing of alternative video synthesis pipelines) and compared its performance to our pipeline by asking blinded participants to evaluate perceptual quality between the two methods. Clinicians preferred videos produced with our pipeline in $80 \pm 11\%$ of cases (binomial test, $p < 0.001$). These findings confirm that the coarse-to-fine approach outperforms a standard GAN in maintaining perceptual quality.

*Contribution of post-processing* Additionally, participants compared our full pipeline, including post-processing between coarse and refine steps, against the exact same pipeline without the post-processing step. Participants preferred videos produced by the full pipeline in $80 \pm 14\%$ of comparisons (binomial test, $p < 0.001$), confirming that the post-processing step adds measurable improvement in perceptual quality.

Overall, the two alternative models exhibit persistent blurriness and artifacts, whereas our full pipeline has better structural and temporal realism. Visual examples are shown in Fig. S3.

Notably, while the gain in perceptual quality is apparent, comparison of SSIM metrics showed no noticeable overall improvement, highlighting the limitations of standard pixel-wise metrics in capturing clinician-perceived quality (see Discussion).

## Discussion

Deep learning has shown potential to be a powerful tool in revolutionizing clinical ultrasound. Nevertheless, the existence of several, often imbalanced sub-modalities can hinder curation of the diverse and balanced datasets needed to develop robust and generalizable DL models for medicine.

Here, we introduce a video-to-video generative translation method that effectively translates two commonly conflated ultrasound sub-modalities, B-mode and CFD. A strength of our study is demonstrated robust performance on diverse ultrasound datasets, including on videos with high proportion of the underlying anatomy obfuscated by CFD signal, requiring significant reconstruction of underlying anatomic structures.

Our findings further illustrate the practical utility of generative image translation in improving the yield, diversity, and class balance of such datasets for use in clinical DL models. It should be noted that the reported impacts of generative translation in improving balance in our clinical imaging dataset are significant underestimates, since, due to the lack of a powerful image translation pipeline previously, annotation of CFD imaging had been systematically avoided to date.

While the main application of the current work is in balancing rare, heterogeneous clinical datasets, the approach is applicable across several imaging modalities and use cases including denoising and super-resolution, highlighting its value for wider research and clinical use.

A/B testing our pipeline against alternative pipelines re-demonstrated the difficulty of this seemingly simple (Fig. 1) video translation task. However, one-step GANs tend to remove the CFD signal without successfully reconstructing underlying anatomy, resulting in artifacts and blurry regions where CFD was originally located. Furthermore, while diffusion models could potentially address these subtle perceptual artifacts, their iterative denoising process presents a prohibitive computational bottleneck for real-time clinical applications. For example, a comparative estimate of our pipeline vs. a state-of-the-art diffusion-based image-to-image translation model^40^ on the NVIDIA L40S GPU used in our Methods shows that our pipeline is approximately 164 times faster than diffusion for video inference (243 milliseconds vs 40 seconds).

We evaluated synthetic videos with several computational and blinded clinician perceptual evaluations, where synthetic medical imaging must be both realistic and clinically accurate. This includes good performance on 11 anatomic structures not included in model training, thus demonstrating the pipeline’s foundational capabilities for video translation.

We found that synthetic videos were statistically indistinguishable from real ones, across computational measures, functional use in deep learning models, and clinician perception. From this cross-functional testing, we also explore a critical issue in the wider field of generative artificial intelligence (AI): that of how best to evaluate AI-generated imaging, especially in high-stakes clinical domains.

Generative models trained in a supervised fashion offer an ability to evaluate instance-to-instance comparisons; in this case, structural similarity between the ground-truth and synthetic B-mode frames. SSIMs between ground truth and synthetic frames were high as an absolute measure and relative to B-mode-CFD and random pairwise B-mode SSIMs (Figs. 3 and 4, Table S2), indicating excellent performance.

Furthermore, synthetic videos were highly realistic in human perceptual testing. Clinicians given a single video could not reliably distinguish whether it was real or synthetic, even when presented with a pair of real and synthetic videos side by side. Additionally, weak to moderate overall correlation between SSIM and human perception was found, but a stronger relationship was observed at lower SSIM values, where visual differences become more pronounced. These findings align with previous literature showing that traditional image quality metrics like SSIM, while widely used in natural imaging tasks, have limits in capturing perceptual and diagnostic quality relevant to clinical interpretation^41,42^.

Although SSIM has recognized limitations in capturing perceptual and diagnostic quality, it remains a widely adopted and interpretable measure of structural similarity and is generally more reflective of perceived image quality than simple pixel-wise metrics such as L1 or L2^41–43^. In this study, SSIM was therefore used alongside additional quantitative metrics and clinician-based perceptual evaluation to provide a more comprehensive assessment of synthetic video realism. Future work may further benefit from incorporating complementary metrics targeting perceptual fidelity and image sharpness, such as deep feature–based perceptual similarity measures (LPIPS) or blur-sensitive quality metrics (CPBD). Additionally, ultrasound-specific validation frameworks, similar to prior studies conducted in magnetic resonance imaging^43^, would help identify the most suitable set of metrics.

We found that our model demonstrated foundational capabilities for image translation since it operated across multiple views and anatomic structures on which it was not trained on, while acknowledging that the definition of a foundation model remains open^13^. The generative model’s ability to translate imaging in ultrasound is already more generalized than the clinical experts asked to judge the model’s outputs; whereas the model operates across adult and fetal use cases, clinical experts are specialized in one area or another.

Although generative translation was excellent overall across several datasets and performance metrics, there were still limitations to our approach. We observed cases where the synthetic videos exhibited noticeable blurriness and occasional artifacts. Thus, perceptual quality remains an area for improvement in our pipeline. This was slightly worse for fetal data compared to adult, reflected in performance metrics. There is a general consensus among experts that fetal imaging is more difficult than adult due to small fetal size and unpredictable motion; furthermore, the use of CFD in the small fetal heart is more likely to encompass the entire heart, covering underlying structures more thoroughly than in adult. Indeed, even on real B-mode video, performance metrics on fetal imaging lagged behind adult; thus, the performance differential between real vs synthetic B-mode data was not statistically significant.

Additionally, the pipeline requires preprocessing with a U-Net to generate CFD masks. Future work can also add additional modules and loss terms in order to learn and attend to CFD regions within the end-to-end pipeline, eliminating the preprocessing step.

With additional training on more, diverse ultrasound data, and with more testing across additional datasets and structures in the future, such a model can serve as a foundation for video translation for ultrasound and could be expanded to further sub-modalities and super-resolution tasks.

## Methods

As shown in Fig. 2, our method employs a coarse network, which produces an initial translation of CFD to B-mode ultrasound which inpaints missing structure, and a refine network, which enhances structural and textural fidelity. The pipeline is trained using pixel-wise, adversarial, and perceptual losses to accurately reconstruct anatomic structures while achieving realistic ultrasound style. We trained and validated on 54,975 videos spanning adult cardiac and fetal ultrasounds.

### Data

De-identified ultrasound imaging from UCSF was used, with waived Informed consent in compliance with the UCSF Institutional Review Board and in accordance with relevant guidelines and regulations. The study was approved by the Ethics committee of the University of California, San Francisco. An ultrasound exam includes numerous videos and images comprising a variety of sub-modalities; for this study, simultaneous acquisitions of both B-mode and CFD sub-modalities–so-called ‘dual’ B-mode/CFD videos–were used for training. We created 10-frame long video snippets, with no overlap. This resulted in 2.25 ± 0.7 (1–15) videos per clip for adult (covering a full cardiac cycle) and 13 ± 10 (1–90) videos per clip for fetal exams (using all available frames).

The adult cardiac ultrasound dataset comprised 21,949 such videos from 982 exams. The fetal ultrasound dataset comprised 41,427 videos from 465 exams; this included both fetal screening ultrasound exams as well as fetal cardiac ultrasound. Both datasets included exams from a range of patients and pathologies, and videos from a range of different views and angles (Table S1). Adult views included: apical two chamber (A2C), apical five chamber (A5C), apical three chamber/long axis (A3C), four chamber (A4C), abdominal aorta or subcostal inferior vena cava (AortaIVC), aortic arch or superior vena cava (AortaSVC), parasternal long axis (PLAX), right ventricular inflow (RVI), right ventricular outflow (RVO), short axis at mid or mitral level (SAX), basal short axis (SAXB), and subcostal four-chamber (SUB4C), as previously described^4^. Fetal views included: three-vessel trachea (3VT), three-vessel view (3VV), left-ventricular outflow tract (LVOT), four chamber (4CH), abdomen (ABDO), and non-target (NT), as previously described^6^. This resulted in an overall dataset of 63,343 videos from 1,447 exams; about 87 percent of videos were used for training/validation (54,975 videos, 1,291 exams), whereas the remaining 13 percent (8,368 videos, 156 exams) were used for testing. Data in the training/validation and test sets did not overlap by video, clip, or exam.

### Data preprocessing

Dual ultrasound RGB frames were split into their constituent B-mode and CFD regions; these were padded to a square aspect ratio and resized to 256x256 pixels using Python’s OpenCV library.

### Estimating CFD masks

To focus the model on CFD regions, we identified and quantified the CFD coverage within each video, using this information to generate binary masks for use in model training (Fig. 2C). A helper U-Net model was trained to segment CFD masks from input CFD frames (Fig. S3). The proportion of foreground pixels in each CFD mask was calculated across the training set and data was accordingly divided into quartiles (Q), serving as a quantitative measure of CFD coverage. CFD coverage was first estimated per-frame and then averaged per video.

### Model architecture and training

We adopt a coarse-to-fine approach as follows, where the coarse network provides inpainting and reconstruction of anatomic structures and the fine network refines video style.

#### Two-stage network

To address the limitations of standard convolutions, which would apply the same filters to both B-mode and CFD pixels, we leveraged gated convolutions^26,28,31^ in the coarse network (Fig. 2A) to adaptively select spatially relevant features^25^. While effective for semantic consistency, gated convolutions suppress high-frequency content, resulting in a blurry output (see coarse example, Fig. 2C)^26,31^.

To enhance recovery of structural details obscured by the CFD signal, we post-processed the coarse output before passing it to the refinement network. This offline step included additive Gaussian noise (0 ± 0.025), stretching the pixel intensity histogram to enhance contrast (Pillow.ImageEnhance.Contrast; factor 1.2), and applying edge-enhancing convolution filters (Pillow.ImageFilter.DETAIL, 2 iterations). Additional edge enhancement was applied at inference time (Pillow.ImageFilter.EDGE_ENHANCE and Pillow.ImageEnhance, factor 1.5) were applied within a blurred version of the CFD mask (cv2.GaussianBlur, kernel 25, sigma 50) prior to the pre-processing steps above (see post-processed coarse example, Fig. 2C).

The refinement network takes as input both the post-processed coarse video and the original CFD video. Both inputs are processed through parallel branches of 3D convolutional blocks with feature exchange. Each convolutional block comprised two convolutional layers followed by instance normalization and a ReLU activation (Fig. 2B).

The number of filters was set to 32–256, and 16–256 range (both doubling at each layer) for coarse and refinement networks respectively; kernel size was 3. MaxPooling was used for downsampling; with size (2,2,2) in the first block and (1,2,2) otherwise, upsampling stride was therefore (2,2,2) in the last convolutional block, and (1,2,2) otherwise. The total number of parameters was 7,738,146 for coarse and 10,905,761 for refinement network.

#### Discriminator

We adopted 3D spatially-strided convolution in the discriminator^28^ (stride size (1,2,2)) with kernel size (3, 5, 5) and LeakyReLU activation function; spectral normalization was implemented. The number of filters was set to 8–64, doubling at each layer (Fig. 2B).

#### Loss function and training

Coarse network was trained using L1 loss for 20 epochs prior to training the refinement GAN. The refinement network was trained with the overall loss function defined as:

1 $$\begin{aligned} L_{\text {total}} = \lambda _{L1} L_{1} + \lambda _{L1mask} L_{1mask} + \lambda _{\text {perceptual}} L_{\text {perceptual}} + \lambda _{\text {adversarial}} L_{\text {adversarial}} \end{aligned}$$

where $\lambda _{L1}=5$, $\lambda _{L1mask}=20$, $\lambda _{\text {perceptual}}=0.1$, and $\lambda _{\text {adversarial}}=1$. Here, $L_1$ is the standard L1 loss applied over all pixels of the video and and $L_{\text {adversarial}}$ is the standard adversarial loss. $L_{1mask}$ is a spatially-weighted L1 loss where CFD masks (see Methods, Estimating CFD masks) assign higher penalties to pixels within CFD regions, emphasizing these areas during training^28,31^. Notably, each 10-frame video is accompanied by a 10-frame mask, with each frame representing its respective CFD region. The perceptual loss, commonly used in image generation tasks to reduce blurriness^25,28,30^, was implemented using the discriminator’s feature representations. Specifically, the L1 distance between discriminator feature representations of real and generated video clips was minimized. Let $\phi _i(\cdot )$ denote the feature map extracted from the *i*-th convolutional layer of the discriminator. The perceptual loss is defined as:

2 $$\begin{aligned} L_{\text {perceptual}} = \sum _i \Vert \phi _i(x, Y) - \phi _i(x, \hat{Y}) \Vert _1, \end{aligned}$$

where *x* is the CFD input, *Y* is the ground-truth video, and $\hat{Y}$ is the generated video. By matching intermediate discriminator activations across all layers, this loss enforces multi-scale structural and texture consistency in both spatial and temporal dimensions.

All weights were determined based on both preliminary experiments and literature. In technical literature, perceptual losses are typically assigned significantly smaller weights^28,30,31^ because they are calculated over high-dimensional feature maps containing numerous high-magnitude activations, whereas $L_1$ losses receive higher weights to ensure that the numerically smaller pixel-wise differences sufficiently guide the model training. Similarly, adversarial losses are typically assigned a default weight of 1 to maintain training stability and a competitive balance between the generator and discriminator.

Training was monitored using the validation SSIM, and it was terminated once SSIM stabilized (reaching 0.93) and the generated videos achieved visually satisfactory quality.

#### Implementation details

The implementation is based on PyTorch 2.6. For each model, training was conducted on an NVIDIA L40S GPU with a batch size of 16. We employed the Adam optimizer with learning rates of 0.0005, 0.0001, and 0.0002 for the coarse, refinement, and discriminator models respectively. Betas were set at 0.9 and 0.999, and half precision was deployed.

#### Data augmentation

We used estimated quartiles of CFD coverage (See Methods, Estimating CFD masks) and assigned weights 1, 2, 3, or 4 to videos with Q1, Q2, Q3, and Q4, respectively, to increase the frequency of videos with higher CFD coverage during training. Additionally, the following data augmentations were applied during training: random rotation within $\pm {10}^\circ$ (p = 0.35), horizontal and vertical flips (p = 0.35 each), random shifts up to 10% (p = 0.35), and random scaling up to 8% (p = 0.35). Gaussian blur (p = 0.35) and additive Gaussian noise (0 ± 0.03, p = 0.35) were applied for CFD input only.

### DL model evaluation of synthetic videos

Previously developed view classification, segmentation models and biometrics for the adult and fetal data, respectively^1,4,6–8^, were used to infer on ground-truth B-mode frames as well as synthetic frames. Of note, these models were trained on real clinical data. The final view classification prediction for each 10-frame video was determined by the most frequently predicted view. The view classifier’s performance was evaluated by the normalized confusion matrix, overall F1 score, and per-class F1 scores. Segmentation performance was evaluated on frames-only, using the Dice scores.

### Blinded clinical expert evaluation of synthetic videos

We selected 200 video examples (ground-truth B-mode video and corresponding synthetic video), each from adult and fetal datasets for evaluation by clinical experts board-certified or board-eligible in adult or fetal ultrasound, respectively (10.9 ± 6.7 years experience, range 1–20). 100 examples were used for each of the following tests. In the first test, blinded clinicians were asked to classify videos by view. Unknown to the clinicians, approximately half the videos were real and half were synthetic. After the fact, clinician performance on real vs synthetic videos was analyzed. All clinicians took the first test.

Then, half the clinicians took the second test and half took the third test. In the second test, clinicians were shown one video and asked to choose if it was real or synthetic. In the third test, clinicians were presented with a pair of videos–the real B-mode video and its synthetic counterpart–and asked to choose the real video. It was only in the second and third tests that the clinicians may have been prompted that the study was about video synthesis (due to the nature of the task).

Finally, the same videos were used in the second test, but this time only synthetic were given to non-blinded subjects (clinical and non-clinical) to evaluate for realism. For each sample, a fool rate coefficient was computed as the proportion of subjects who perceived it as real. We then calculated the correlation between the fool rate and the SSIM scores comparing B-mode and synthetic images.

Of note, it is known that GANs are poorly suited for generating text and fiducial markings^44,45^. Therefore, we replaced these elements in the synthetic outputs with the corresponding fiducials from the real B-mode data. We used the Segment Anything Model (SAM-Huge^46^) to detect and extract the fiducials.

### A/B testing of alternative video synthesis pipelines

To evaluate the impact of our pipeline’s coarse-to-fine approach, we implemented a one-step GAN inspired by well-established image-to-image translation frameworks^34^ as a comparator. This GAN shared our primary generator and discriminator architectures (Fig. 2B, only CFD input to Generator) and was optimized via a combination of $L_1$ and adversarial loss, where both loss components were assigned equal weights. To test the pipeline without the image post-processing step, we simply utilized the identical architecture and training hyperparameters as the full pipeline, with the exception that the generated coarse videos were not subjected to the post-processing before refinement stage.

### Statistical testing

Statistical tests were performed using the two-sample, non-parametric Mann-Whitney U (MWU) test except where a different test is mentioned. Effective number of groups (sites, classes) was calculated as e to the power of the Shannon entropy across groups.

## Supplementary Information

Supplementary Information.

## Data Availability

Due to the sensitive nature of patient data, we are not able to make these data publicly available at this time. Code will be made available upon publication. The Corresponding Author is the point of contact.
