# Supplementary material for: Generative deep learning for foundational video translation in ultrasound
Source: Sci Rep. 2026 Apr 16;16:17586. doi: 10.1038/s41598-026-47777-z (PMC13243462; doi:10.1038/s41598-026-47777-z)
Supplement: Supplementary file 1 — Supplementary Information. [file 41598_2026_47777_MOESM1_ESM.pdf]

Supplemental Results

Estimating temporal consistency of synthetic videos

Temporal consistency was quantified using the mean warping error. For each video, dense optical flow was estimated between consecutive frames using Gunnar Farneback's algorithm[1] (cv2.CalcOpticalFlowFarneback). Frame t was then spatially warped into the coordinate system of frame $t+1$ using the computed flow fields. The temporal consistency error was calculated as the mean absolute error (MAE) between the warped frame and the actual target frame, measured in grayscale intensity units (0–255). A lower score indicates higher temporal stability and a reduction in flickering artifacts.

We compared the temporal coherence of synthetic and real videos by analyzing the distributions of mean temporal consistency error. The synthetic videos achieved a mean error of 4.84 ± 2.63, which closely aligns with that of real videos at 4.89 ± 2.43. No statistically significant difference between the two groups ($p = 0.16$, Welch's t-test) was observed, suggesting that the temporal stability of the synthetic videos is comparable.
Furthermore, we visualized the Kernel Density Estimation (KDE) to compare the error distributions of both datasets. This analysis confirms that the synthetic videos closely mirror the temporal characteristics of the ground truth, with overlapping density peaks and standard deviation intervals, indicating high-quality motion synthesis without major artificial flickering.

Helper U-Net

To generate binary masks of the CFD-containing regions, we trained a helper U-Net to detect the CFD region from each frame. We leveraged the subset of dual B-mode-CFD frames (Fig. S4A) to obtain usable labels for the CFD region only (Fig. S4D). We then trained a custom U-Net with CFD (Fig. S4A) as input and the prepared labels (n=1120) as outputs. Dilation-erosion operations (OpenCV version 4.11.0.86, dilate, erode) were used to remove any residual color doppler box. The final CFD masks are shown in Fig. 2C.

Supplemental Figures

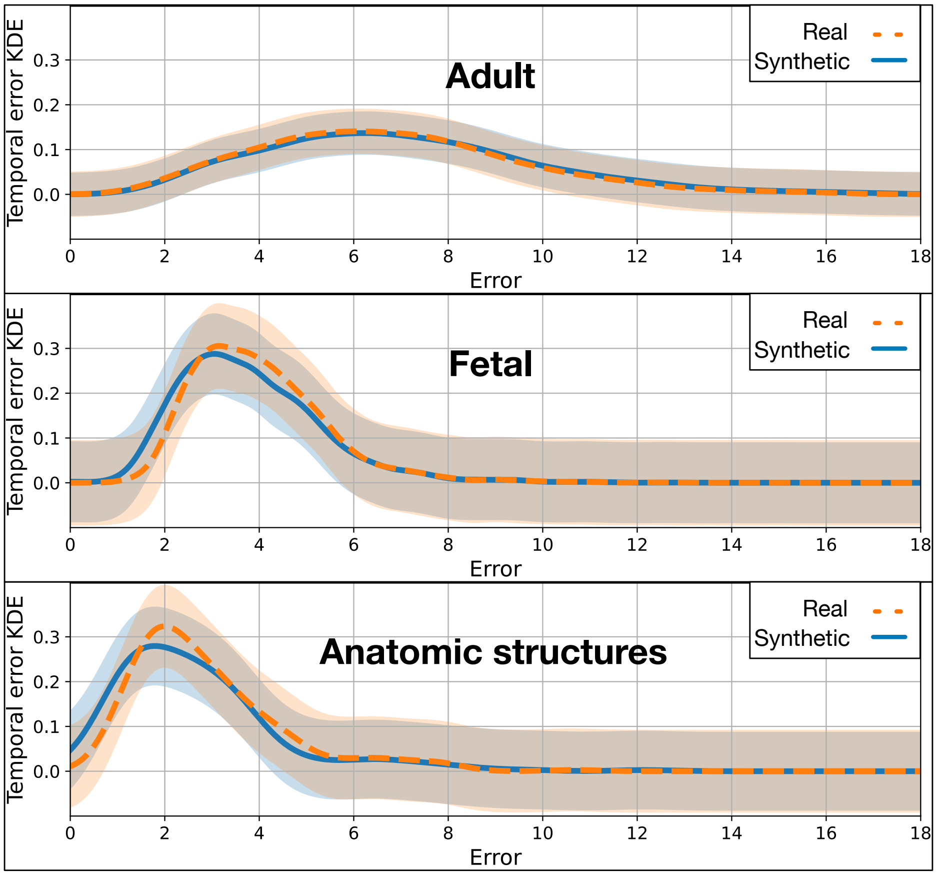

Figure S1. Temporal consistency analysis of real vs. synthetic videos. Kernel Density Estimation (KDE) of the temporal warping error for synthetic (solid, blue) and real (dashed, orange) videos; shaded regions represent the standard deviation. Synthetic videos closely approximate the temporal profile of the real videos in all test datasets: (A) adult, (B) fetal, and (C) anatomic structures.

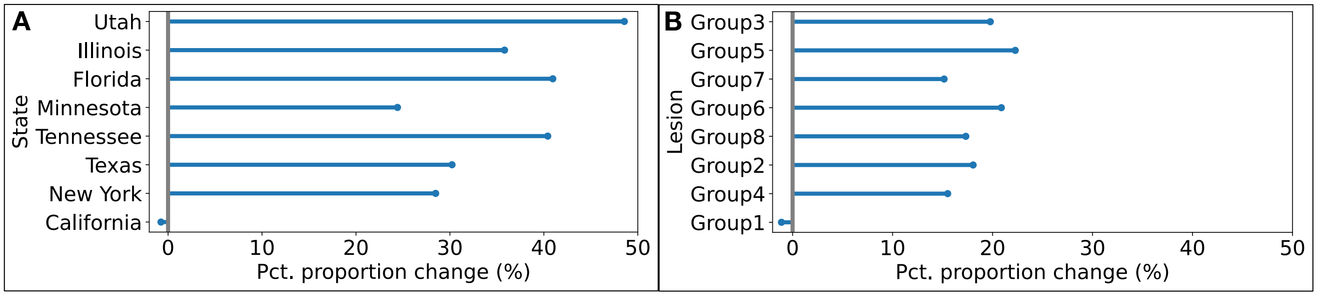

Figure S2. Impact of synthetic data on fetal dataset balance, precisely number of clips. (A) Percentage change in class proportions for U.S. states and (B) clinical lesion groups. Categories are ordered by baseline prevalence (least to most represented). Positive values indicate increased proportional representation, demonstrating a reduction in dataset imbalance. Lesion groups (1–8) are categorized by clinical pathology. Group 1 includes normal hearts; Group 2 includes hypoplastic left heart syndrome (HLHS), single ventricle, left atrial isomerism (LAI), right atrial isomerism (RAI), and tricuspid atresia; Group 3 includes levo-transposition of the great arteries (LTGA) and total anomalous pulmonary venous return (TAPVR); Group 4 includes tetralogy of Fallot (TOF), dextro-transposition of the great arteries (DTGA), truncus arteriosus, and double outlet right ventricle (DORV); Group 5 includes Ebstein anomaly; Group 6 includes aortic stenosis and pulmonary atresia with intact ventricular septum (PAIVS); Group 7 includes atrioventricular septal defect (AVSD); and Group 8 includes coarctation of the aorta.

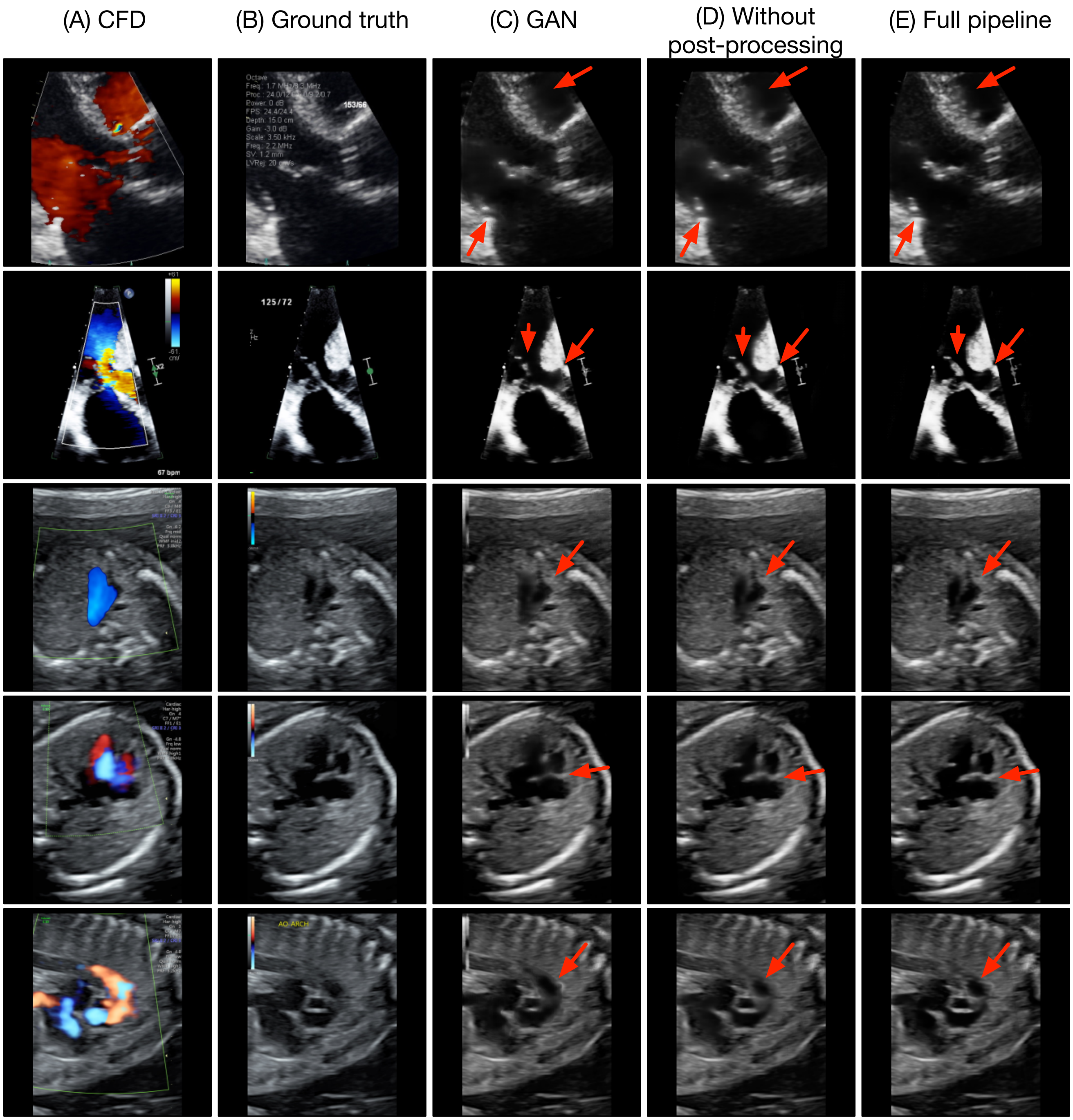

Figure S3. Visual comparison of different generation strategies. Arrows point to presence of artifacts and lack of high frequency detail in (C) and (D) compared to our proposed model (E) and real images (B).

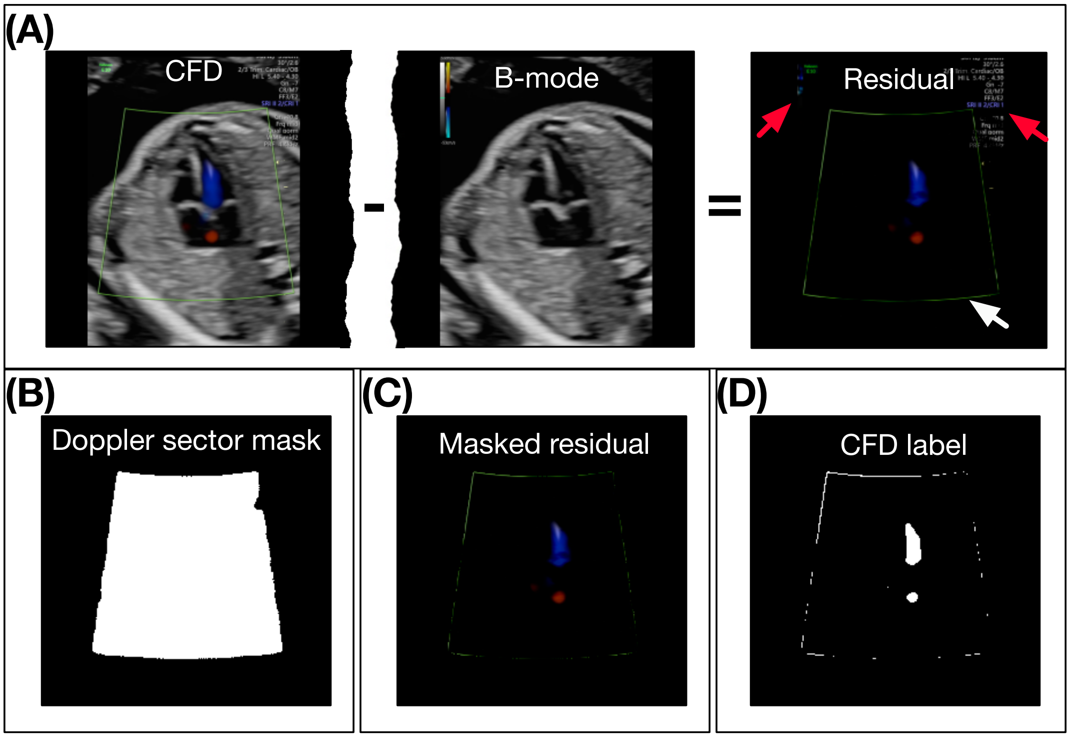

Figure S4. Generating training labels for helper U-Net. (A) From a dual (CFD/B-mode) acquisition, B-mode is subtracted from CFD and the residual image is kept, which includes the Doppler sector (white arrow) and fiducial markings (red arrows). (B) The Doppler sector is segmented using the Segment Anything Model (SAM-Huge[2]). (C) The residual image from (A) is then masked and only the CFD-containing region is kept. (D) Thresholding is applied to generate usable labels for the CFD-containing region.

Supplemental Tables

View	Train Videos	Train Exams	Validation Videos	Validation Exams	Test Videos	Test Exams
A2C	765 (4.8%)	267 (7.1%)	138 (4.7%)	56 (8.0%)	137 (4.8%)	49 (7.2%)
A5C	1093 (6.8%)	323 (8.5%)	196 (6.7%)	58 (8.2%)	243 (8.5%)	64 (9.4%)
A3C	733 (4.6%)	237 (6.3%)	145 (4.9%)	44 (6.2%)	149 (5.2%)	47 (6.9%)
A4C	2878 (17.9%)	431 (11.4%)	553 (18.8%)	84 (11.9%)	557 (19.4%)	82 (12.1%)
AortaIVC	1796 (11.2%)	486 (12.9%)	335 (11.4%)	92 (13.1%)	282 (9.8%)	78 (11.5%)
AortaSVC	1316 (8.2%)	352 (9.3%)	275 (9.3%)	72 (10.2%)	241 (8.4%)	58 (8.5%)
PLAX	1120 (7.0%)	292 (7.7%)	189 (6.4%)	50 (7.1%)	241 (8.4%)	55 (8.1%)
RVI	671 (4.2%)	245 (6.5%)	105 (3.6%)	43 (6.1%)	89 (3.1%)	39 (5.7%)
RVO	41 (0.3%)	21 (0.6%)	8 (0.3%)	5 (0.7%)	9 (0.3%)	4 (0.6%)
SAX	786 (4.9%)	304 (8.0%)	134 (4.5%)	54 (7.7%)	147 (5.1%)	62 (9.1%)
SAXB	3081 (19.1%)	447 (11.8%)	536 (18.2%)	76 (10.8%)	523 (18.3%)	80 (11.8%)
SUB4C	1825 (11.3%)	375 (9.9%)	333 (11.3%)	70 (9.9%)	246 (8.6%)	61 (9.0%)
**Unique adult**	**16105**	**728**	**2947**	**134**	**2864**	**120**
3VT	1485 (5.2%)	164 (13.5%)	396 (5.3%)	36 (13.3%)	314 (5.7%)	24 (14.8%)
3VV	2207 (7.8%)	198 (16.3%)	429 (5.7%)	43 (15.9%)	488 (8.9%)	24 (14.8%)
LVOT	4466 (15.7%)	233 (19.2%)	1325 (17.7%)	53 (19.6%)	929 (16.9%)	29 (17.9%)
4CH	3091 (10.9%)	216 (17.8%)	913 (12.2%)	49 (18.1%)	1139 (20.7%)	32 (19.8%)
ABDO	348 (1.2%)	73 (6.0%)	119 (1.6%)	22 (8.1%)	85 (1.5%)	18 (11.1%)
NT	16830 (59.2%)	329 (27.1%)	4314 (57.6%)	67 (24.8%)	2549 (46.3%)	35 (21.6%)
**Unique fetal**	**28427**	**358**	**7496**	**71**	**5504**	**36**
**Total unique**	**44532**	**1086**	**10443**	**205**	**8368**	**156**

Table S1. Training, validation, and test datasets showing the number and percentage of videos and unique exams per clinical view, along with totals for the adult and fetal datasets. Videos correspond to 10-frame video snippets, and exams correspond to clinical ultrasound examinations. Note that the total number of unique exams is not the sum across views, since a single exam may contribute to multiple views. A2C – apical two chamber, A5C – apical five chamber, A3C – apical three chamber/long axis, A4C/4CH – four chamber, AortaIVC – abdominal aorta/inferior vena cava, AortaSVC – aortic arch/superior vena cava, PLAX – parasternal long axis, RVI – right ventricular inflow, RVO – RV outflow, SAX – short axis, SAXB – basal short axis, SUB4C – subcostal four-chamber, 3VT – three-vessel trachea, 3VV – three-vessel view, LVOT – left-ventricular outflow tract, ABDO – abdomen, NT – non-target.

**Dataset / Quartile**	**Synthetic vs real SSIM** mean ± std (range)	**CFD vs real SSIM** mean ± std (range)	**Wasserstein distance**
ADULT Q1	0.93 ± 0.03 (0.82, 0.97)	0.88 ± 0.04 (0.71, 0.94)	0.05
ADULT Q2	0.93 ± 0.03 (0.82, 0.96)	0.87 ± 0.04 (0.70, 0.93)	0.06
ADULT Q3	0.91 ± 0.03 (0.83, 0.96)	0.83 ± 0.04 (0.68, 0.91)	0.07
ADULT Q4	0.87 ± 0.05 (0.35, 0.95)	0.78 ± 0.06 (0.33, 0.87)	0.09
**OVERALL ADULT**	**0.91 ± 0.04 (0.35, 0.97)**	**0.84 ± 0.06 (0.33, 0.94)**	**0.07**
FETAL Q1	0.92 ± 0.08 (0.10, 0.98)	0.91 ± 0.08 (0.11, 0.97)	0.01
FETAL Q2	0.93 ± 0.03 (0.10, 0.98)	0.91 ± 0.03 (0.11, 0.95)	0.02
FETAL Q3	0.93 ± 0.02 (0.83, 0.96)	0.89 ± 0.02 (0.78, 0.94)	0.03
FETAL Q4	0.90 ± 0.03 (0.69, 0.96)	0.85 ± 0.04 (0.62, 0.92)	0.05
**OVERALL FETAL**	**0.92 ± 0.04 (0.10, 0.98)**	**0.88 ± 0.05 (0.11, 0.97)**	**0.03**
OTHER Q1	0.92 ± 0.02 (0.87, 0.97)	0.93 ± 0.02 (0.87, 0.98)	0.01
OTHER Q2	0.92 ± 0.02 (0.84, 0.95)	0.91 ± 0.03 (0.77, 0.96)	0.01
OTHER Q3	0.91 ± 0.04 (0.59, 0.94)	0.89 ± 0.04 (0.56, 0.94)	0.02
OTHER Q4	0.89 ± 0.06 (0.43, 0.95)	0.85 ± 0.07 (0.41, 0.92)	0.04
**OVERALL OTHER**	**0.91 ± 0.05 (0.43, 0.97)**	**0.89 ± 0.06 (0.41, 0.98)**	**0.02**
**OVERALL ALL**	**0.91 ± 0.04 (0.10, 0.98)**	**0.87 ± 0.06 (0.11, 0.98)**	**0.04**

Table S2. SSIM performance by amount of CFD signal per video.

Structure	Test videos	Test Exams
Extremity	11	8
Abdominal	15	9
Procedural	2	1
Pelvic	3	2
Soft Tissue	2	1
Neck	2	1
Testicular	5	3
Chest	1	1
Neurologic	32	17
Pancreatic	147	45
Renal	40	23
**Total count**	**260**	**111**

Table S3. Additional test data from a range of anatomies not seen during training.

References

1. Farnebäck, G. Two-frame motion estimation based on polynomial expansion. In *Image Analysis*, 363–370, DOI: 10.1007/3-540-45103-x_50 (Springer Berlin Heidelberg, Berlin, Heidelberg, 2003).
2. Kirillov, A. *et al.* Segment anything, DOI: 10.48550/arXiv.2304.02643 (2023). ArXiv:2304.02643.
